# Politics and Medicine

**Published:** 1856-10

**Authors:** 


### *Politics and Medicine.*

"*Eloquence and Poetry—Dr. Holmes on Sergery and Sumner.*—At the late Annual Festival of the Massachusetts Doctors, Dr. Holmes, in speaking of the dignity and usefulness of the healing art, thus very happily alludes to Mr. Sumner:

"Nay, come down to nearer times and places, and look into the chamber where our own fellow citizen, struck down without warning by the hand of brutal violence, lies prostrate, and think what fearful issues hang on the skill or incompetence of those who have his precious life in charge. One little error, and the *ignis sacar*, the fiery plague of the wounded, spreads its angry blush over the surface, and fever and delirium are but the preludes of deadlier symptoms. One slight neglect, and the brain, oppresed with the products of disease, grows dreamy, and then drowsy; its fine energies are palsied, and too soon the heart that filled it with generous blood is stilled forever. It took but a little scratch from a glass broken at his daughter's wedding, to snatch from life the great anatomist and surgeon, Spigelius, almost at the very age of him whose recovery we look to not without anxious solicitude.

"At such an hour as this, more than at any other, we feel the dignity, the awful responsibility of the healing art. Let but that life be sacrificed and left unavenged, and the wounds of that defenceless head, like the foul witch's blow on her enchanted image, are repeated on the radiant forehead of Liberty herself, and flaw the golden circlet we had vainly written with the sacred name of Union!

"Dii, prohibite minas! Dii talem avertite casum!

"I give you, Mr. President,

"The Surgeons of the City of Washington—God grant them wisdom, for they are dressing the wounds of a mighty empire and of uncounted generations."

Dr. Holmes's sentiment was received by the rising of the whole Society, who responded with three hearty and enthusiastic cheers."—*American Medical Gazett.*

"*Senator Sumner and the Doctors.*—The newspaper accounts of the wounds of the Senator of Massachusetts, and the professional treatment to which he has been subjected, have so mystified his case as to render it ludicrous if it were not obvious that they are furnished by reporters for partizan presses, whose ignorance and prejudices conceal the truth. Dr. Bunting, of Montreal, who is itinerating through the country, with the celebrated curiosity of the man with an open wound in his stomach, on exhibition to the Faculty, seems to have been the first surgeon who saw the wound, and he swears that it was upon his head, and that it was some six inches *deep!* which, if true, would have brained the Senator, and been "past all surgery." Next we have the evidence of several other medical men, essentially differing from each other as to the nature and extent of the injury, and so widely, that we cannot decide whether the wounds are trivial or serious, and whether his absence from his seat was from necessity or choice, and this point has even been mooted in the Senate.

These conflicting statements and their source do not concern us at all, but from our professional stand-point we cannot forbear criticising his treatment in the light of surgical science, and this irrespective of the medical men concerned. That he was not a martyr seems to us to have been marvellous, and we congratulate him and his assailant on his extraordinary escape.

First, then, if the wounds were made through the scalp by blows with a cane, these wounds were incised, but lacerated, and the adjacent tissues contused. Stitches were therefore contra-indicated, and ought not to have been used; nor were we surprised at the bulletins announcing the erysipelas which followed such bad surgery, and endangered the patient's life.

Secondly—That the blows, as described by all parties, produced very severe concussions of the brain, and this was by far the greatest source of hazard to the Senator's life. Now, according to surgical rule, a dark room, a silent chamber, the prohibition of all conversation, and the exclusion of company, should have been enjoined and enforced; thus withdrawing the stimuli of light, sound, mental effort, and society, and absolute rest should have been insisted on as the first condition of recovery. Instead of this course being taken, his brain was speedily taxed to call up the circumstances and witnesses of his attempted assassination, and he was subjected to an inquisitorial examination on these topics. Had his surgeons done their duty to their patient, they would have resisted this jeopardy to his life, though urged by reasons of State, by appealing to a "higher law;" and, if necessary, had they employed a "gutta percha cane" upon the heads of the intruders, it would have been in proof that they were true to their patient, and we could have forgiven them much easier than we can Mr. Brooks. On the whole, we regard the recovery of Mr. Sumner to be an extraordinary escape."—*American Medical Gazette.*

One of the noblest impulses of man's nature is that which prompts him to overlook the faults of his fellow men, or even to throw a veil over the nakedness of their sinning. But there are times when truth and justice so loudly call for their victim, that even kind and officious charity shrinks from the clamor, and willingly leaves him stripped before the blast of public scorn and contempt.

What high-minded and honorable son of Æsculapius can read the above effusions without the deepest sensations of shame, pity and contempt? Shame, that he should find himself affiliated by a common name with the authors; pity, that humanity should be so weak as to bend thus under the yoke of an ever changing public opinion; contempt, that anything wearing breeches and calling itself a man, should allow its mind to sink so far below the level of propriety and professional decency as to make that press, which it has sworn to devote to the noble cause of science a mere vehicle for hauling away the dirty garbage from a political metropolis.

Think, ye men of science, of the Annual Meeting of the Massachusetts Medical Society being the scene of political fanaticism! Think of the "whole society" rising to give three cheers to the little physic-poet, who had not only volunteered his cacoethic muse against the "brutal violence" of a Southern representative, but had shaped her into an empty, vain-glorious threat against the peace of our common land! Think of a body of scientific men, meeting to cheer the apostate who could thus wantonly debauch science whilst pretending to espouse her chastity! And then think of the editor of a medical journal, which claims to be the exponent of, and champion for those high principles which should govern professional gentlemen, stepping aside from his obvious and sfficiently onerous duties to lend his pen to slanderous tales of "attempted assassination," and wanton insults to medical men, who from their "stand-point" of professional reputation, might safely dare him to the scale of public opinion! Think of an editor branding as "bad surgery"

the treatment of a case, from which he was so far removed, and of the nature of which he in plainest words acknowledges himself to be *ignorant!*

Did we think that the conduct of these men would elicit any other sentiment than the scorching censure of all good and true men, we would devote a few pages to the record of overwhelming evidence in favor of "the Surgeons of the City of Washington;" but every heart that is free from the poison of fanaticism, every heart that soars above pandering to leprous appetites, every heart that knows no geographical or political boundary to science, all *noble* hearts, will throb condemnation of the spirit which prompted them to speak and write.

That such men will live in history is most improbable, unless the Pythagorean doctrine be true, and in the transmigration of souls they are found in the shape of the ruthless little moth, preying on the valuable pages, as they would now prey on the fair reputation of their fellow men.

Alas! that it should have come to this; that Medicine at the North is to be drawn up in hostile array against Medicine at the South; that science is to droop under the withering influence of a political fanaticism which has seized the least virtuous portion of our people! Shall it be so? No, our very ink refuses to flow to the record of such a suggestion, and it blushes to write down the names of the rebels this day found in our ranks. In the borrowed language of the physic-poet—

*Dii, prohibite minas! Dii, talem avertite cnsum!*

[*N. O. Med. News & Hosp. Gaz.*

